# Trends in Transmission of Drug Resistance and Prevalence of Non-B Subtypes in Patients with Acute or Recent HIV-1 Infection in Barcelona in the Last 16 Years (1997-2012)

**DOI:** 10.1371/journal.pone.0125837

**Published:** 2015-06-03

**Authors:** Juan Ambrosioni, Omar Sued, David Nicolas, Marta Parera, María López-Diéguez, Anabel Romero, Fernando Agüero, María Ángeles Marcos, Christian Manzardo, Laura Zamora, Manuel Gómez-Carrillo, José María Gatell, Tomás Pumarola, José María Miró

**Affiliations:** 1 Hospital Clínic-Institut d’Investigacions Biomèdiques August Pi-Sunyer (IDIBAPS), University of Barcelona, Barcelona, Spain; 2 Huésped Foundation, Buenos Aires, Argentina; 3 Agency for Health Quality and Assessment of Catalonia (AQuAS), Barcelona, Spain; 4 Department of Microbiology. Barcelona Centre for International Health Research (CRESIB) Hospital Clínic, Barcelona, Spain; 5 Institute for Biomedical Research in Retroviruses and AIDS, Buenos Aires, Argentina; University of Athens, Medical School, GREECE

## Abstract

**Objectives:**

To evaluate the prevalence of transmitted drug resistance (TDR) and non-B subtypes in patients with acute/recent HIV-1 infection in Barcelona during the period 1997-2012.

**Methods:**

Patients from the “Hospital Clínic Primary HIV-1 Infection Cohort” with a genotyping test performed within 180 days of infection were included. The 2009 WHO List of Mutations for Surveillance of Transmitted HIV-1 Drug Resistance was used for estimating the prevalence of TDR and phylogenetic analysis for subtype determination.

**Results:**

189 patients with acute/recent HIV-1 infection were analyzed in 4 time periods (1997-2000, n=28; 2001-4, n=42; 2005-8, n=55 and 2009-12, n=64). The proportion of patients with acute/recent HIV-1 infection with respect to the total of newly HIV-diagnosed patients in our center increased over the time and was 2.18%, 3.82%, 4.15% and 4.55% for the 4 periods, respectively (p=0.005). The global prevalence of TDR was 9%, or 17.9%, 9.5%, 3.6% and 9.4% by study period (p=0.2). The increase in the last period was driven by protease-inhibitor and nucleoside-reverse-transcriptase-inhibitor resistance mutations while non-nucleoside-reverse-transcriptase inhibitor TDR and TDR of more than one family decreased. The overall prevalence of non-B subtypes was 11.1%, or 0%, 4.8%, 9.1% and 20.3 by study period (p=0.01). B/F recombinants, B/G recombinants and subtype F emerged in the last period. We also noticed an increase in the number of immigrant patients (p=0.052). The proportion of men-who-have-sex-with-men (MSM) among patients with acute/recent HIV-1 infection increased over the time (p=0.04).

**Conclusions:**

The overall prevalence of TDR in patients with acute/recent HIV-1 infection in Barcelona was 9%, and it has stayed relatively stable in recent years. Non-B subtypes and immigrants proportions progressively increased.

## Introduction

Antiretroviral therapy (ART) has dramatically changed the natural history of HIV infection. Most naïve patients who begin ART today suppress viral replication and achieve functional restoration of the immune system. However, during treatment, almost one-quarter of patients experience virological failure and often have resistant HIV isolates [1]. The widespread use of ART and the increased survival of patients receiving it make the transmission of resistant HIV strains likely to occur. Resistant strains have been reported in infections acquired through sexual contact, vertical transmission and exposure to infected blood [2, 3]. Thus, transmitted drug resistance (TDR) has become a relevant public health problem. Active surveillance of TDR provides important information about the factors involved in the transmission of resistant HIV strains and in the selection of ART components. It plays a major role in the design of strategies to control the evolution and emergence of resistance [4].

Worldwide, the prevalence of resistance in acute or recent HIV-1 infections ranges from 5% to 24.5%[2, 5]. In Spain, the multicenter studies performed have very small samples [6, 7], and there are no previous reports from Barcelona.

HIV-1 subtype B infections have traditionally been predominant among the infected European population, particularly in men-who-have-sex-with-men (MSM). However, the prevalence of non-B subtypes is increasing in developed countries, as result of international travel and population migration [8, 9].

The aims of this study were to estimate the prevalence of antiretroviral resistance mutations and non-B subtypes in a cohort of 189 consecutive patients with acute or recent HIV-1 infection in a tertiary teaching hospital in Barcelona, Spain, and to describe the pattern of changes over a 16-year period (1997–2012).

## Materials and Methods

### Study population

The study population comprised patients from the “Hospital Clinic Primary HIV-1 Infection Cohort” consecutively evaluated within 180 days after HIV infection at the Hospital Clínic, Barcelona, Spain, between January 1, 1997 and December 31, 2012. The inclusion criteria were detectable viremia with a negative HIV serology result or documented seroconversion within the 6 months prior to the first evaluation. In symptomatic patients with several exposures, the date of infection was assumed to be 14 days before the beginning of symptoms. For asymptomatic seroconverters, the date of infection was assumed to be the midpoint between the last negative test result and the first positive one. At the time of genotyping, patients with an estimated time of infection of less than 30 days were defined as ‘acute infection’ and those with an estimated time of infection between 30 and 180 days as ‘recent infection’. Patients with resistance tests performed beyond 180 days after the suspected day of infection were excluded from the analysis.

Patients were classified into 4 periods according to the year of diagnosis: 2009–2012 (widespread availability in Barcelona of 4 new drugs: darunavir, etravirin, raltegravir and maraviroc) and three earlier periods of equal duration: 1997–2000, 2001–2004 and 2005–2008.

### Virological analyses

HIV serology was determined using a microparticle enzyme immunoassay (AxSYM, Abbott Laboratories, Illinois, USA) and confirmed by line immunoassay (Inno-LIA HIV I/II Score, Innogenetics, Ghent, Belgium). Viremia was measured using the Cobas Amplicor Monitor (Roche Molecular Systems, Branchburg, New Jersey, USA) or the Versant HIV-1 RNA 1.0 Assay kPCR (Siemens Healthcare, Erlangen, Germany) with a limit of detection of 50 or 37 copies/mL, respectively. Genotypic mutations of both the reverse transcriptase gene and the protease gene from viral RNA were detected using the ViroSeq HIV Genotyping System v.2 (Abbott Laboratories, Illinois, USA) and an ABI3100 sequencer. HIV- 1 subtype characterization was first performed by using the REGA HIV-1 Subtyping Tool of the Stanford database (available from http://dbpartners.stanford.edu/RegaSubtyping/) and confirmed by Neighbor-Joining (NJ) phylogenetic analyses by using the MEGA version 6 program [10]. NJ phylogenetic trees were inferred under the Kimura 2-parameter (K2-P) nucleotide substitution model and reliability of the obtained tree topology was estimated with the bootstrap method with reference sequences obtained from the HIV Sequence Database, Los Alamos National Laboratory (LANL; www.hiv.lanl.gov). HIV-1 sequences suspected to be recombinants in the NJ phylogenetic tree were analyzed by bootscan analyses with Simplot 3.5.1 software [11]. The amino acid substitutions selected by highly active antiretroviral therapy (HAART) and associated with drug resistance were identified using the 2009 World Health Organization (WHO) list of mutations for surveillance of drug resistance [12]. Drug mutations for integrase inhibitors were not analyzed since they are not routinely performed for patients with primary infection in our institution. Mutations not included in the 2009 WHO list, but associated with resistance to rilpivirine according to Stanford HIV drug resistance database were also reported due to the clinical relevance.

### Statistical analysis

The chi-square test or the Fisher exact test was used, as appropriate, to compare categorical variables, and the Mann-Whitney or Klustal-Wallis tests were used, as appropriate, to compare continuous variables. Links between resistant genotype and sex, age, risk factors, symptoms, CD4 and CD8 cell counts, and viral load were tested. All p values were considered significant at <0.05. As described above, 4 time periods were analyzed: 1997–2000, 2001–4, 2005–8 and 2009–12. The correlation between the increase in non-B HIV-1 subtypes and the increase in immigration during the 4 study periods was evaluated using Spearman’s Rank Correlation Coefficient. All statistical analyses were performed using SPSS software, version 17.

The study was approved by the Institutional Review Board (Hospital Clínic-Institut d’Investigacions Biomèdiques August Pi-Sunyer-IDIBAPS-). All patients signed the informed consent form.

## Results

### Patient characteristics according to analyzed periods

During the study period, 5,109 newly diagnosed patients underwent a first evaluation at our center; of these, 199 (3.89%) met the criteria for primary HIV infection (PHI) and were enrolled in the “Hospital Clínic Primary HIV-1 Infection Cohort”. Ten patients were excluded due to the genotypic resistance test being performed beyond 180 days post-infection; thus 189 patients were included in the final analysis. Baseline characteristics of the whole population studied and according to periods are described in Table 1: 92.6% were male, median (interquartile range) age was 33 (28–39) years; for 81%, the main route of transmission was sexual relations between MSM, and 31.2% were immigrants, most of whom from Latin America (56%), followed by other Western European countries (15%) and Eastern European countries (10%). There were no patients from Sub-Saharan Africa. According to the predefined analyzed periods, 28 patients were included in 1997–2000, 42 in 2001–4, 55 in 2005–8 and 64 in 2009–12. The proportion of patients with PHI out of the total of newly HIV-diagnosed patients increased over the time and was 2.18%, 3.82%, 4.15% and 4.55% for the 4 periods, respectively (p = 0.005).

**Table 1:** Baseline characteristics of patients and study periods.

		Period				
	total	1997–2000	2001–2004	2005–2008	2009–2012	p
N (%)	189 (100)	28 (14.8)	42 (22.2)	55 (29.1)	64 (33.9)	
Gender*						0.011
Male	175 (92.6)	26 (92.9)	34 (81)	53 (96.4)	62 (96.9)	
Female	14 (7.4)	2 (7.1)	8 (19)	2 (3.6)	2 (3.1)	
Age** (n = 188)	33 (28–39)	30 (26–36)	31 (27–37)	34 (28–38)	34 (27–39)	0.275
<30*	63 (33.5)	13 (46.4)	16 (38.1)	14 (25.5)	20 (31.7)	
30–40	82 (43.6)	10 (35.7)	20 (47.6)	27 (49.1)	25 (39.7)	
40–50	33 (17.6)	3 (10.7)	4 (9.5)	12 (21.8)	14 (22.2)	
>50	10 (5.3)	2 (7.1)	2 (4.8)	2 (3.6)	4 (6.3)	
Route of transmission*						0.004
MSM-bisexual	153 (81)	21 (75)	30 (71.4)	47 (85.5)	55 (85.9)	
Heterosexual	21 (11.1)	2 (7.1)	8 (19)	6 (10.9)	5 (7.8)	
IDU	10 (5.3)	5 (17.9)	4 (9.5)	1 (1.8)	0 (0)	
Unknown	5 (2.6)	0 (0)	0 (0)	1 (0)	4 (6.3)	
Origin*						0.052
Native	120 (63.5)	24 (85.7)	24 (57.1)	36 (65.5)	36 (56.3)	
Immigrant	59 (31.2)	2 (7.1)	14 (33.3)	18 (32.7)	25 (39.1)	
Unknown	10 (5.3)	2 (7.1)	4 (9.5)	1 (1.8)	3 (4.7)	
Symptomatic*						0.626
yes	162 (85.7)	23 (82.1)	34 (81)	49 (89.1)	56 (87.5)	
no	27 (14.3)	5 (17.9)	8 (19)	6 (10.9)	8 (12.5)	
Plasma HIV-1 log10RNA** (n = 188)	5.17 (4.51–5.80)	5.04 (4.39–5.80)	5 (4.43–5.40)	5.45 (4.75–5.62)	5.41 (4.50–5.89)	0.168
<5.0*	78 (41.5)	13 (46.4)	21 (50)	19 (35.2)	25 (39.1)	
>5.0	110 (58.5)	15 (53.6)	21 (50)	35 (64.8)	39 (60.9)	
CD4 cell count/ul** (n = 188)	494 (375–619)	494 (376–637)	584 (438–740)	506 (377–618)	402 (309–562)	0.009
<350	45 (23.9)	6 (21.4)	8 (19)	10 (18.5)	21 (32.8)	
350–500	52 (27.7)	8 (28.6)	8 (19)	15 (27.8)	21 (32.8)	
>500	91 (48.4)	14 (50)	26 (61.9)	29 (53.7)	22 (34.4)	
Acute or Recent infection at genotyping*						0.21
Acute (infection of <30 days)	23 (12.2)	2 (7.2)	1 (2.4)	9 (16.4)	11 (17.2)	
Recent (infection between 30 and 180 days)	166 (87.8)	26 (92.8)	41 (97.6)	46 (83.6)	53 (82.8)	
Resistant strain (any mutation)*	17 (9)	5 (17.9)	4 (9.5)	2 (3.6)	6 (9.4)	0.2
Non-B subtypes*	20 (10.6)	0 (0)	2 (4.8)	5 (9.1)	13 (20.3)	0.01

* n(%)

** median (IQR)

MSM: men-who-have-sex-with-men

IDU: injective drug user

### Genotypic drug resistance

**Fig 1:**
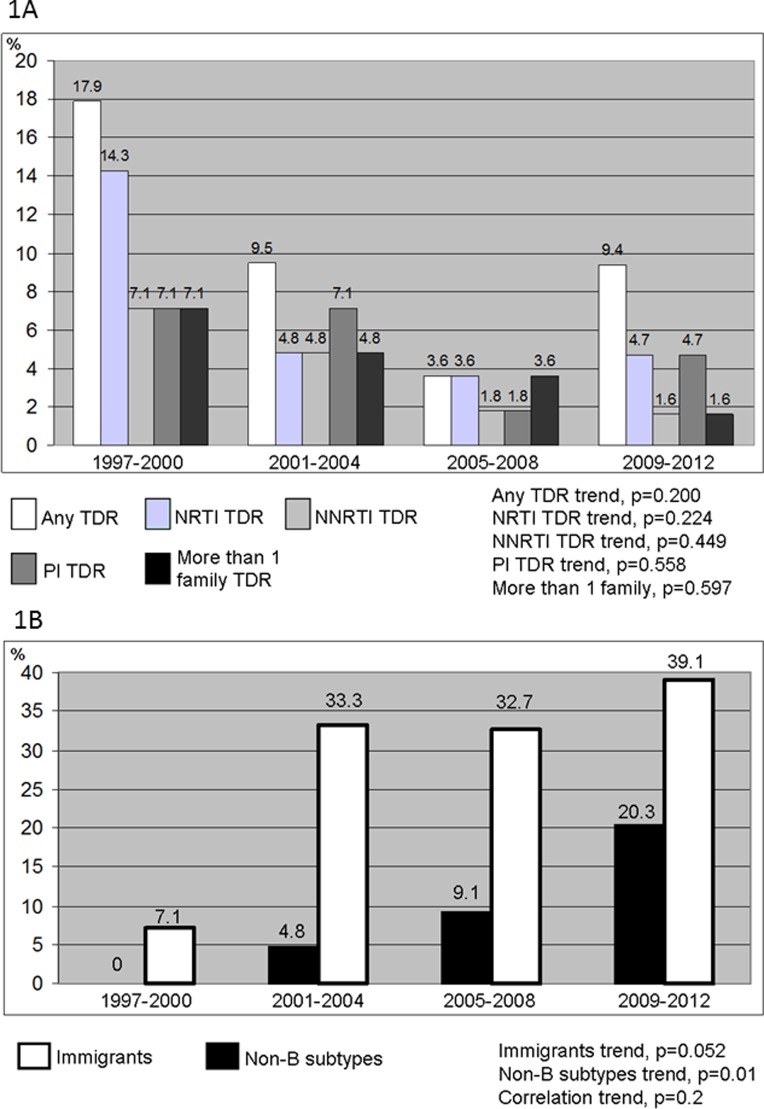
Fig 1A: Prevalence of transmitted drug resistance mutations by study period and drug family according to the 2009 WHO list. Fig 1B: Prevalence of non-B HIV-1 subtypes and immigrants by study period. Footnote figure: NRTI indicates nucleoside reverse transcriptase inhibitor; NNRTI, non-nucleoside reverse transcriptase inhibitor; PI, protease inhibitor.

According to the WHO list of mutations, 9% of patients had a strain with 1 or more resistance mutations. The prevalence of TDR in patients with acute or recent HIV-1 infection decreased until the period 2005–8, and slightly increased in 2009–12. The proportion of resistance was 17.9% for the period 1997–2000, 9.5% for the period 2001–4, 3.6% for the period 2005–8 and 9.4% for the period 2009–12 (p = 0.2). The increase in the last period was found in nucleoside-reverse-transcriptase-inhibitor (NTRI) associated mutations (3.6% in 2005–8 and 4.7% in 2009–12) and in protease inhibitor (PI) associated mutations (1.8% in 2005–8 and 4.7% in 2009–12). Non-nucleoside reverse transcriptase inhibitor (NNRTI) associated resistance mutations, and resistance mutations to more than one family, however, decreased in the last period (Fig 1A). Overall, there were no differences in rates of TDR between the MSM and the heterosexual population (9.2% and 9.5% respectively, p = 0.628). There was no TDR among injection-drug-users (IDUs).

On the basis of the WHO proposed mutations, NRTI mutations were found in 11 patients (5.8%), NNRTI mutations were found in 6 patients (3.2%) and PI mutations were found in 9 patients (4.8%). Resistance to at least 2 drugs from the different families was observed in 7 patients (3.7%). Some resistance mutations, such as Q151M or K65R, were not found. Details of mutations found in each period are shown in Table 2. The E138A mutation, not listed by WHO 2009 list but associated to rilpivirine resistance was detected in 4 patients, 3 in the 2005–2008 period and 1 in the 2009–2012 period. No other rilpivirine associated mutation was detected.

**Table 2:** Details of mutations found according to antiretroviral family and period of study.

		Period			
Mutations N (%)*	total	1997–2000	2001–2004	2005–2008	2009–2012
	189	28	42	55	64
**NRTI**					
M41L	6 (3.2)	2 (7.1)	2 (4.8)	0 (0)	2 (3.1)
D67N	3 (1.6)	1 (3.6)	0 (0)	1 (1.8)	1 (1.6)
T69N	2 (1.1)	1 (3.6)	0 (0)	0 (0)	1 (1.6)
K70R	2 (1.1)	2 (7.1)	0 (0)	0 (0)	0 (0)
M184V	1 (0.5)	1 (3.6)	0 (0)	0 (0)	0 (0)
L210W	3 (1.6)	1 (3.6)	1 (2.4)	1 (1.8)	0 (0)
T215F/Y	3 (1.6)	2 (7.2)	1 (2.4)	0 (0)	0 (0)
T215S/D/L	2 (1.1)	0 (0)	0 (0)	2 (3.6)	0 (0)
K219E/Q	4 (2.2)	2 (7.2)	0 (0)	1 (1.8)	1 (1.6)
**NNRTI** ^**&**^					
K101E	1 (0.5)	0 (0)	1 (2.4)	0 (0)	0 (0)
K103N	3 (1.6)	1 (3.6)	1 (2.4)	0 (0)	1 (1.6)
Y181C	3 (1.6)	1 (3.6)	1 (2.4)	1 (1.8)	0 (0)
G190A	1 (0.5)	0 (0)	1 (2.4)	0 (0)	0 (0)
**PI**					
M46I/L	5 (2.7)	2 (7.1)	2 (4.8)	0 (0)	1 (1.6)
I54L	1 (0.5)	1 (3.6)	0 (0)	0 (0)	0 (0)
V82A/F/I/T	7 (3.7)	3 (1.6)	2 (4.8)	0 (0)	2 (3.1)
L90M	2 (1.1)	0 (0)	1 (2.4)	1 (1.8)	0 (0)

* Only mutations found in at least one case according to WHO list of TDR are listed.

^&^ E138A mutation, not listed by WHO 2009 list but associated to rilpivirine resistance was detected in 4 patients (2.1%), 3 (5.4%) in the 2005–2008 period and 1 (1.6%) in the 2009–2012 period.

NRTI: Nucleoside/nucleotide reverse transcriptase inhinitors

NNRTI: Non-nucleoside reverse transcriptase inhibitors

PI: Protease inhibitors

### HIV-1 Subtypes

**Fig 2:**
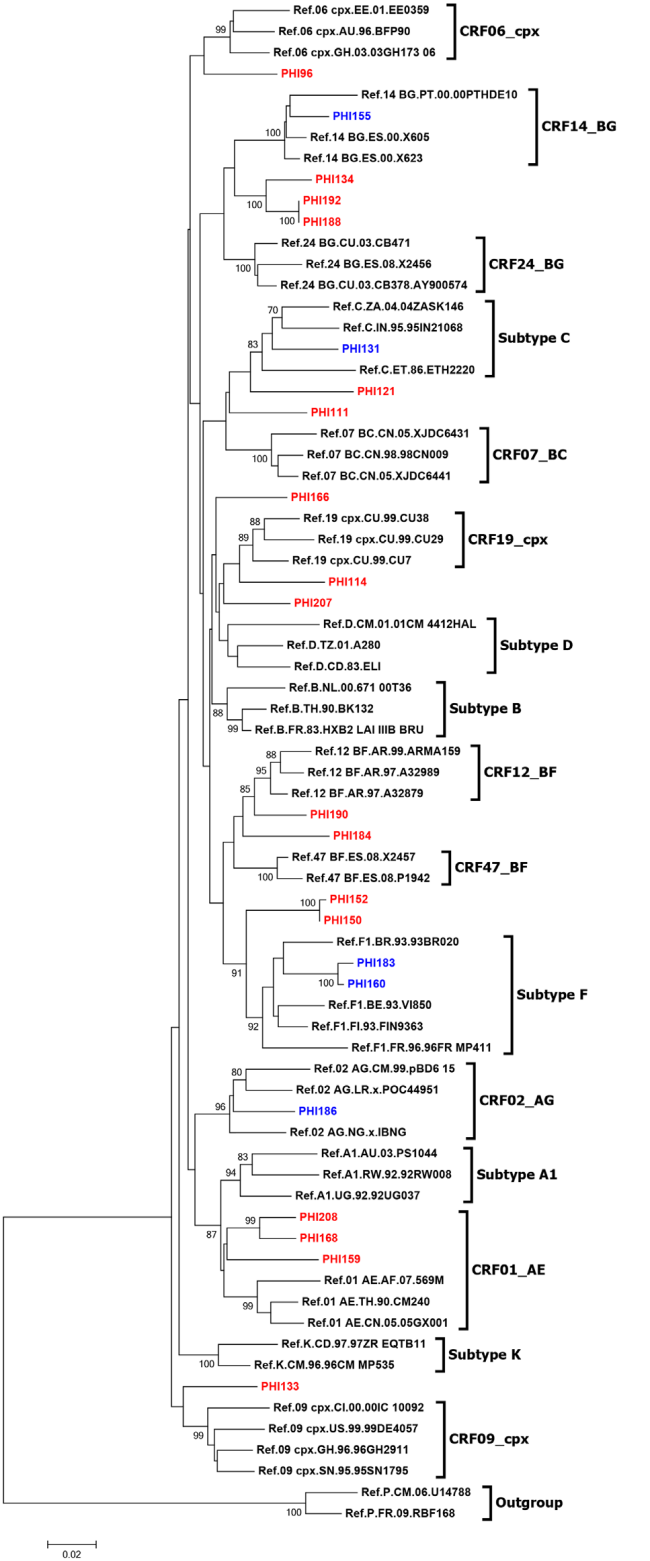
Phylogenetic tree analysis of the non-B HIV-1 pol sequences. Footnote figure: The phylogenetic inferences were performed by the Neighbor-Joining algorithm under the Kimura-2 parameter nucleotide substitution model with bootstrap. HIV-1 pure subtype and CRFs reference sequences were obtained from Los Alamos National Laboratory (LANL; www.hiv.lanl.gov). Bootstrap values of 70% or greater provide reasonable confidence for genotype assignment (sequences represented in blue). Sequences represented in red were suspected to be recombinants and re-analyzed by bootscanning (data not shown).

Non-B subtypes were identified in 20 cases (10.6%). HIV-1 non-B subtype distribution increased over the 4 periods: 0%, 4.8%, 9.1% and 20.3%, respectively, with a clear linear trend (p = 0.01, Fig 1B). Both immigrant and non-B subtype proportions increased over the time (Spearman’s Rank Correlation Coefficient = 0.8, p = 0.2). Non-B subtypes included subtypes A, n = 1; C, n = 2; F, n = 2; G, n = 1; A/G recombinant, n = 1; B/G recombinants, n = 2; B/F recombinants, n = 3; CRF01_AE, n = 2; and other recombinants (B/C recombinant, B/CRF06_cpx, CRF19_cpx (D segment), CRF02_AG/CRF09_cpx, CRF14_BG, CRF02_AG/Subtype B recombinant), n = 6 (Fig 2). All the Subtype F, B/G recombinants and B/F recombinants were reported in the last period (2009–2012).

The proportion of males was comparable between patients with subtype B and non-B (92.9% vs. 90%; p = 0.647), but regarding the route of transmission, MSMs were significantly higher among patients with B subtype (82.8% vs. 65%) and heterosexuals among those with non-B subtypes (20% vs. 10.1%; p = 0.001). There were no IDUs infected with non-B subtypes. The proportion of immigrants was not different among patients infected with B and non-B subtypes (30.8% and 35% respectively, p = 0.524). Patients infected by a non-B subtype were slightly older (36 vs. 33 years old) and age categories differently distributed (p = 0.037). Finally, TDR mutations were found in similar proportions among patients infected with subtype B and non-B strains (8.9% and 10% respectively, p = 0.697). A comparison of patients infected with B and non-B subtypes is shown in Table 3.

**Table 3:** Patients characteristics according to subtype of infection (B or non-B).

	total	B subtype	Non-B subtypes	p
N (%)	189 (100)	169 (89.4)	20 (10.6)	
Gender*				0.647
Male	175 (92.6)	157 (92.9)	18 (90)	
Female	14 (7.4)	12 (7.1)	2 (10)	
Age** (n = 188)	33 (28–39)	33 (28–39)	36 (29–47)	0.037
<30*	63 (33.5)	58 (34.3)	5 (26.3)	
30–40	82 (43.6)	77 (45.6)	5 (26.3)	
40–50	33 (17.6)	27 (16)	6 (31.6)	
>50	10 (5.3)	7 (4.1)	3 (15.8)	
Route of transmission*				0.001
MSM-bisexual	153 (81)	140 (82.8)	13 (65)	
Heterosexual	21 (11.1)	17 (10.1)	4 (20)	
IDU	10 (5.3)	10 (5.9)	0 (0)	
Unknown	5 (2.6)	2 (1.2)	3 (15)	
Origin*				0.524
Native	120 (63.5)	107 (63.3)	13 (65)	
Immigrant	59 (31.2)	52 (30.8)	7 (35)	
Unknown	10 (5.3)	10 (5.9)	0 (0)	
Symptomatic*				1
yes	162 (85.7)	145 (85.8)	17 (85)	
no	27 (14.3)	24 (14,.2)	3 (15)	
Plasma HIV-1 log10RNA** (n = 188)	5.17 (4.51–5.80)	5.17 (4.60–5.80)	5.50 (4.89–5.92)	0.533
<5.0*	78 (41.5)	71 (42.3)	7 (35)	
>5.0	110 (58.5)	97 (57.7)	13 (65)	
CD4 cell count/ul** (n = 188)	494 (375–619)	490 (380–614)	512 (372–619)	0.175
<350	45 (23.9)	39 (23.2)	6 (30)	
350–500	52 (27.7)	50 (29.8)	2 (10)	
>500	91 (48.4)	79 (47)	12 (60)	
Resistant strain (any mutation)*	17 (9)	15 (8.9)	2 (10)	0.697

* n(%)

** median (IQR)

MSM: men-who-have-sex-with-men

IDU: injective drug user

## Discussion and Conclusions

In this study, the prevalence of TDR for antiretroviral drugs in patients with acute or recent HIV-1 infection (less than 6 months) in a single reference center in Barcelona over a period of 16 years (1997–2012) was 9%. In a meta-analysis including 26 studies performed in Spain [13], those performed between 1996–2003 reported prevalence values of TDR that decreased from 26.7% to 6.7%. This wide range can be explained by differences in methodology. However, data from the studies performed between 2004–8 showed a narrower range: 11% to 2.9%[13]. De Mendoza et al. showed a decrease in the prevalence of resistance in PHI in other Spanish cities from 20% in 1999 to 3.4% in 2001 [14]. The authors claimed that these changes were due to the decrease in the number of patients with detectable viremia due to HAART and to the increase in new infections transmitted by immigrants from areas with no access to ART (many of whom were carrying HIV-1 non-B infections)[14]. However, access to ART has increased in developing countries. This is consistent with our results up to 2008, but we noticed a new increase in overall TDR in the last period (2009–12), although this increase was only seen for some antiretroviral families (PI and NRTI), while NNRTI TDR and multi-drug resistance transmission decreased. However, the increased single PI resistance mutations have very limited clinical consequences, considering the high genetic barrier of these drugs. A boosted-PI regimen is the regimen of choice when ART needs to be initiated in a patient with PHI and where the resistance test is still unavailable [15]. Moreover, we found the E138A mutation, associated with reduced susceptibility to rilpivirine in 4 patients of our cohort. This is related to the polymorphic nature of the mutation, since it was found in patients infected years before the the drug became available. Indeed, prompt initiation of ART during PHI has not only clinical but also epidemiological consequences: PHI patients are a significant source of HIV transmission [16]. Thus early therapy may decrease transmissibility [15–17].

While some studies suggest that the prevalence of TDR mutations was decreasing in Spain until 2009 [18], data published by Yebra et al.[19] for 354 HIV-1 infected patients diagnosed between 1999 and 2007 in 4 Spanish HIV/AIDS clinics (3 in Madrid and 1 on the Canary Islands) seems to contradict those results. The overall prevalence of TDR was 13.8%, and the authors did not observe a decreasing temporal trend, but an increasing one. Moreover, they found a significantly higher prevalence of NNRTI resistance mutations among patients infected by non-B subtypes [19]. This must be considered in the context of the expansion of ART access in developing countries, in which NNRTI-based regimens (particularly efavirenz) are the most frequently prescribed. The most recent results of the CoRIS Spanish Cohort found 7.9% of TDR according to the WHO list of mutations [20], which is consistent with our results.

The results of studies from France and Italy also showed a decrease until the early/mid-2000s [21, 22]. However, in Germany and in France in more recent studies, TDR seems to be stabilizing to levels comparable to our report in the last period (2009–12)[23, 24]. In Europe, the most important data sets come from the SPREAD Programme, which prospectively investigates TDR among patients with newly diagnosed HIV-1 infection in 20 European countries and Israel. Data from 1996–2002 showed a prevalence of 13.5% among recently infected patients [25]. During the following study period, 2002–5, the prevalence seems to be stabilizing at 8.4%[26]. In a more recently published, large multi-cohort European study, TDR showed a rate of around 10%[27]. These values are comparable with the 9% of global TDR found in our study, and with the 9.4% of the last period.

Surveys in the United States report an increase in the prevalence of TDR rates during recent years. In 2005 surveys, the TDR rates were 25%[5], while previous reports showed a rate of 8.3% for 1997–2001 (26). Little et al. reported an increase from 3.4% in 1995–8 to 12.4% in 1999–2000 in 10 US cities [28]. The National HIV Surveillance system reported TDR rates of 14.6% in 2006 [29] and 16% in 2007 for 10 states and 1 county in the US [30]. In a more recent study performed in New York City in patients with acute or recent infection (median 66.5 days), TDR prevalence was 14.3%[31]. Rates can be influenced by different definitions and methodologies, but resistance could also vary according to demographic factors and access to health care and antiretroviral treatment. The US Surveillance study performed in 2006 showed that the prevalence of mutations varied with ethnicity and risk behavior: 14% in white MSM compared with less than 5% in Hispanic or African-American heterosexual men or African-American women [32], probably due to differences in access to health care in the US [33]. In Catalonia, the health care system is universal and free and provides ART to all those who need it, reducing the impact of these differences. In our cohort, we did not observe resistance in IDUs. Possible explanations are that IDUs are being infected by individuals not exposed to ART and that this transmission route is becoming less frequent. Indeed, there were no patients with PHI who had acquired the HIV infection through injection use in the last period (2009–12) of our study. However, the descriptive design and the small number of patients in this study prevent us from drawing definite conclusions for this risk group of patients.

Resistance mutations may persist for a variable time following transmission [34, 35]. Nevertheless, NRTI-associated mutations can gradually disappear, particularly those reducing viral fitness (such as M184V). A study performed in the framework of the Swiss HIV Cohort Study found that M184V minority variants were present in 8.2% of acute/recently infected patients and only in 2.5% of chronic/established infections [36]. Thus, the timing of the genotypic test may also explain differences in detected rates both in European and American studies.

Non-B subtype infections account for 20–40% of new HIV diagnoses in some European countries [37–39]. It is noteworthy that, in our study, no cases of infection by these HIV-1 non-B variants were reported in 1997, and they then progressively increased with time. A retrospective Spanish study performed outside Catalonia from 1995 to 2003 found that non-B subtypes were recognized in 43.2% of HIV-1-infected subjects with epidemiological suspicion of infection by non-B subtypes [40]. In another Spanish study including 198 seroconverters from different cities outside Catalonia, between 1997 and 2004, the frequency of non-B subtype HIV infections was only 7.6%, and no cases were found before 2002 [6]. Another region with high rates of non-B HIV infections is Galicia (a province with predominantly Portuguese and African immigration)[41]. Indeed, Pernas et al. recently reported a 37.8% of non-B variants in northwest Spain, and subtype F was the most prevalent. These patients may also have and impaired virological response [42]. In our report, we found an increasing trend over time of non-B subtype HIV infections in Barcelona. B/F recombinants, B/G recombinants and subtype F were exclusively found in the last period. The MSM population, which represented almost 90% of the cases of PHI in our study in the last period (2009–12), seems to be particularly affected by this emergence. Indeed, although MSM was the most prevalent risk factor in all periods, the proportion among the total patients with PHI increased over time, suggesting a clear issue of transmission in early phases of HIV infection in this group. A correlation was observed between the increase in immigration and the increase in non-B subtypes. Therefore, it may be hypothesized that immigrants are infected locally by B subtypes, whereas Spanish-born patients become infected by non-B subtypes during sexual contact with immigrants infected by non-B variants.

Our study has several limitations. First, the limited number of patients infected by intravenous drug use limits the extrapolation of our conclusions. Second, the fact that some mutations are identified as resistant does not necessarily indicate clinical resistance. Last, the number of patients included per period progressively increased. A small bias in selection criteria for recent infection (within the 180-day period) cannot completely been excluded, which might have reduced the detection of some resistance mutations in a determined period. The study does, however, have several strengths. The number of patients with PHI included is very large for a single-center study. It has been performed over a long period of time, allowing us to analyze also epidemiological and risk trends in a longitudinal way.

Antiretroviral resistance is a frequent, dynamic, and complex phenomenon that should be considered from both individual and public health perspectives. The heterogeneous nature of data might be due to temporal and geographic variations, differences in the resistance tests used, timing of sampling, local prevalence of HIV subtypes, and scarce access to health care. It is necessary to establish a consensus regarding the definition of a resistance mutation and recent HIV infection in order to ensure the quality and comparative value of data.

In conclusion, the overall prevalence of resistant HIV-1 strains in acute and recent HIV-1-infected patients in Barcelona was 9%. Non-B subtypes are emerging in our population and the proportion of immigrants among patients with acute or recent HIV infection is increasing. These data should be taken into account when starting combined antiretroviral therapy in this setting and in the development of strategies for the prevention of HIV transmission, particularly in groups such as MSM.

## Supporting Information

S1 File Transmission of drug resistance and Non-B HIV-1 subtypes dataset. (XLS)
